# Common Shared Pathogenic Aspects of Small Vessels in Heart and Brain Disease

**DOI:** 10.3390/biomedicines10051009

**Published:** 2022-04-27

**Authors:** Rita Moretti, Milijana Janjusevic, Alessandra Lucia Fluca, Riccardo Saro, Giulia Gagno, Alessandro Pierri, Laura Padoan, Luca Restivo, Agnese Derin, Antonio Paolo Beltrami, Paola Caruso, Gianfranco Sinagra, Aneta Aleksova

**Affiliations:** 1 Department of Internal Medicine and Neurology, Neurological Clinic, 34100 Trieste, Italy; moretti@units.it (R.M.); paola.caruso@asugi.sanita.fvg.it (P.C.); 2 Cardiothoracovascular Department, Azienda Sanitaria Universitaria Giuliano Isontina (ASUGI) and University of Trieste, 34100 Trieste, Italy; mjanjusevic@units.it (M.J.); alessandralucia.fluca@units.it (A.L.F.); riccardo.saro@asugi.sanita.fvg.it (R.S.); giulia.gagno@studenti.units.it (G.G.); alessandro.pierri@studenti.units.it (A.P.); luca.restivo2@studenti.units.it (L.R.); agnese.derin@asugi.sanita.fvg.it (A.D.); gianfranco.sinagra@asugi.sanita.fvg.it (G.S.); 3 Cardiology and Cardiovascular Physiopathology, Azienda Ospedaliero-Universitaria S. Maria Della Misericordia, 06156 Perugia, Italy; laura.padoan@ospedale.perugia.it; 4 Department of Medicine (DAME), University of Udine, 33100 Udine, Italy; antonio.beltrami@uniud.it

**Keywords:** small-vessel disease, microvascular dysfunction, coronary-artery disease, ischemic-heart disease, myocardial infarction with non-obstructive coronary arteries (MINOCA), atrial fibrillation, subcortical-vascular dementia, stroke, cerebral-amyloid angiopathy, blood–brain barrier, microglial activation, nitric oxide, endothelial dysfunction, inflammation, C-reactive protein (CRP), interleukin (IL-6), tumor-necrosis-factor α (TNF-α), amyloid β-peptide

## Abstract

Small-vessel disease (SVD), also known as microvascular endothelial dysfunction, is a disorder with negative consequences for various organs such as the heart and brain. Impaired dilatation and constriction of small vessels in the heart lead to reduced blood flow and ischemia independently of coronary artery disease (CAD) and are associated with major cardiac events. SVD is usually a silent form of subcortical vascular burden in the brain with various clinical manifestations, such as silent-lacunar-ischemic events and confluent white-matter hyperintensities. Imaging techniques are the main help for clinicians to diagnose cardiac and brain SVD correctly. Markers of inflammation, such as C-reactive protein, tumor-necrosis-factor α, and interleukin 6, provide insight into the disease and markers that negatively influence nitric-oxide bioavailability and promote oxidative stress. Unfortunately, the therapeutic approach against SVD is still not well-defined. In the last decades, various antioxidants, oxidative stress inhibitors, and superoxide scavengers have been the target of extensive investigations due to their potential therapeutic effect, but with unsatisfactory results. In clinical practice, traditional anti-ischemic and risk-reduction therapies for CAD are currently in use for SVD treatment.

## 1. Introduction

**Figure 1:**
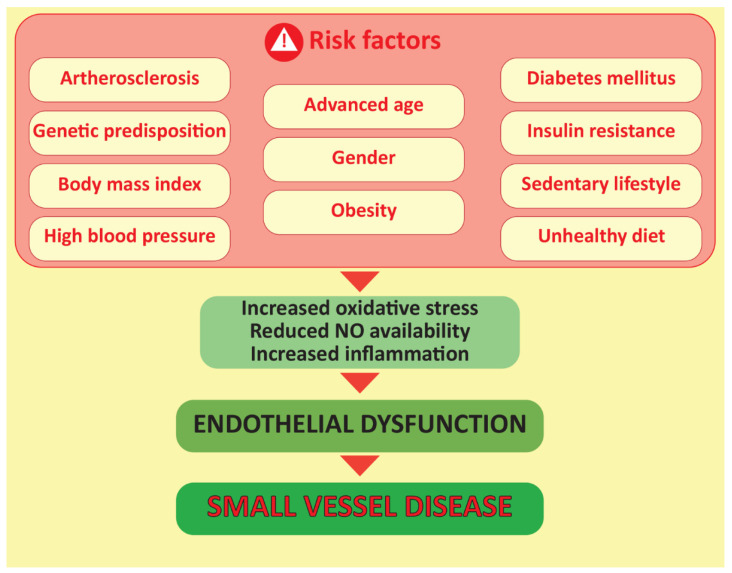
The figure describes the negative effects of a number of vascular risk factors that cause oxidative stress, reduced nitric-oxide (NO) production and the promotion of an inflammatory state that later leads to endothelial dysfunction underlying small-vessel disease. NO, nitric oxide.

Small-vessel disease (SVD) consists of multiple pathological processes that affect small arteries, arterioles, venules, and capillaries, and, therefore, influences the function of various organs such as the heart and brain [1]. Vascular risk factors such as age, gender, high blood pressure, body-mass index (BMI), diabetes mellitus, insulin resistance, obesity, a sedentary lifestyle, an unhealthy diet, and genetic predisposition to cardiovascular disease trigger various pathophysiological processes that lead to endothelial dysfunction, which underlies SVD (Figure 1) [2]. Impaired vasodilation of coronary microcirculation leads to inadequate blood flow and ischemia, which can occur independently of the presence of obstructive-coronary-artery disease (CAD). Unfortunately, not infrequently, SVD is associated with cardiovascular events and poor outcomes [2]. In the brain, there are many consequences of this silent killer, such as major vascular occlusion (stroke) and subcortical vascular dementia (sVAD) [2]. Worth mentioning here is that, pathophysiologically, the heart and the brain share some similarities, and the main one is that the vascular anatomy is quite similar, with arteries on the surface and penetrating arteries that provide tissue perfusion [3].

It is important to emphasize that, due to the aging of the population, it has recently been noticed that SVD affects the heart and brain more often [3,4]. Unfortunately, there is limited data and very little knowledge on this subject. For example, the epidemiology of large blood vessels and the effect on various organs has been thoroughly researched and documented, while the epidemiology of SVD and its consequences on the heart [3] and on the brain [4,5,6] is less well established. After 2003, many clinical reports began to pay special attention to SVD, which led to an exchange of experiences between experts and the implementation of joint research projects and successful therapies.

The review aims to gather current knowledge on the pathogenesis of SVD and possible links between cerebrovascular and cardiovascular complications. We would especially like to focus on pathophysiological processes that lead to endothelial dysfunction and available diagnostic and prognostic biomarkers that can give us insight into the disease, diagnostic tools used in clinical practice, and therapeutic possibilities. In addition, we would like to point out gaps in knowledge that could encourage future studies.

## 2. Heart Small-Vessel Disease, or, Better Said, Coronary Microvascular Dysfunction?

Cardiac SVD is commonly known as coronary-microvascular disease, microvascular-endothelial dysfunction, or coronary-microvascular dysfunction (CMD). CMD is a condition that includes numerous structural and functional mechanisms that seriously alter coronary microcirculation’s physiological function, leading to the impairment of dilation and constriction of the small vessels in the heart [7,8]. Consequently, reducing the oxygen-rich blood flow to the heart causes ischemic heart disease [7,8]. The actual prevalence of CMD is currently challenging to assess. The incidence of the disease is estimated to be even higher than expected, as nonspecific symptoms and limited diagnostic testing make the development of a diagnostic algorithm difficult [9,10]. A CMD diagnosis is often demanding, since its clinical presentation is shared with symptoms of obstructive CAD; for example, chest pain, often coupled with dyspnoea, is the most common initial symptom [9,10]. Furthermore, both CMD and CAD share the same risk factors, such as smoking, diabetes mellitus, and dyslipidemia [11].

The most well known cause of ischemic heart disease is obstructive CAD. Nevertheless, it is estimated that approximately 40% of patients with acute myocardial infarction are negative for obstructive atherosclerosis [11], meaning that the myocardial ischemia could be only partially explained by plaque formation. While epicardial coronary arteries (diameter 5 mm–500 μm) offer low resistance and are prone to atheroma formation, coronary microcirculation is not involved in plaque development [12]. As coronary microcirculation is directly responsible for regulating blood flow in response to myocardial oxygen demand, there is growing interest in how functional and structural alterations impact myocardial ischemia [13]. It is noteworthy that, according to the current clinical classification of CMD, microcirculation alterations in the absence of CAD or myocardial diseases are only one of the four manifestations of the disease. The other classes consist of CMD with myocardial diseases, obstructive CAD, and iatrogenic dysfunction [13].

A recent study on patients with myocardial infarction with non-obstructive coronary arteries (MINOCA) showed the coexistence of functional anomalies in response to vasodilatory or vasoconstrictor stimuli in small peripheral arteries, including the brain [14]. Another example is the evidence that patients with atrial fibrillation (AF) have a higher prevalence of cerebral SVD and cognitive impairment, regardless of cardioembolic stroke [15]. The correlation between AF and the rapid development of cerebral-ventricular dilatation and white-matter lesions in the elderly cohort has already been demonstrated [15]. Furthermore, patients with AF often present the same risk factors associated with SVD, such as arterial hypertension, old age, diabetes mellitus, and obesity [15]. In addition, in a recent article, in a cohort of patients with AF, most of whom were adequately anticoagulated, it was found that 5.5% of the subjects developed new cerebral infarcts in the two-year follow-up and that these correlated with a cognitive deficit. This fact suggests how anticoagulant therapy alone is not sufficient to prevent new brain infarcts in all cases of AF [16]. Finally, some interesting data emerged among patients with COVID-19, which resulted in a higher risk of developing cardiovascular and cerebrovascular disease in the post-acute phase [17,18]. This association appears to be related to the inflammatory endothelial cell damage and long-lasting hyperactivated immune response [17,18].

### Physiopathology of Coronary-Microvascular Dysfunction

The pathology of SVD in the heart focuses on the pre-arterioles (diameter 500–100 μm), arterioles (diameter <100 μm) and capillaries (diameter <10 μm) [12,13]. Endothelium dysfunction is the core of CMD, since endothelium sustains cardiovascular homeostasis by controlling various processes such as blood-fluid maintenance, regulation of vascular tone, permeability to nutrients and metabolites, modulation of the inflammatory response, and coagulation balance [2,19]. The regulation of pre-arterioles’ diameter is a response to blood flow and intravascular pressure that stimulate endothelial cells to produce vasoactive mediators such as nitric oxide (NO) and endothelin, which mediate vessel relaxation and contraction by acting on vascular-smooth-muscle cells (VSMCs) [12]. On the other hand, myocardial metabolites such as adenosine, carbon dioxide, and hydrogen peroxide, directly control arterioles’ vasomotor regulation due to their position and wall thickness [20,21].

In the case of CMD not associated with obstructive CAD or primary and secondary cardiomyopathies, microvascular alterations are mediated by risk factors that promote endothelial dysfunction through inflammation and NO deficiency [13,19]. In addition to impairing vessel contractility, endothelial dysfunction increases adhesion protein expression, permeability, and oxidative stress [19]. In the case of CMD with obstructive CAD, as expected, the presence of obstructive atherosclerosis is initially responsible for sustaining endothelial dysfunction, and it is also the cause of changes in blood flow downstream of the occlusion site, which affects the compensatory capacity of pre-arterioles and contributes to CMD [13,22]. The vasomotor alterations of microvessels cause coronary spasms, which manifest as chest pain [21]. In CMD, with or without CAD, inflammation and endothelial dysfunction are also responsible for structural abnormalities, including vessel remodeling and a decrease in capillary density [12]. Specifically, the alteration of NO availability, cyclic guanosine monophosphate (cGMP), and growth factors, such as the transforming growth factor-beta (TGF-β), mediate the VSMCs proliferation and the conversion of endothelial cells into fibroblasts, which produce collagen [23]. In the media layer of microvasculature, these mechanisms lead to luminal narrowing and increased wall thickness [24], which impair the vasomotor response to myocardial metabolites. Furthermore, capillary rarefaction due to insufficient growth factors reduces oxygen delivery to the myocardium [23]. It is known that structural changes occurring in CMD associated with primary myocardial diseases, such as dilated and hypertrophic cardiomyopathy, increase the risk of adverse events [24,25].

## 3. Cerebral-Small-Vessel Disease

Cerebral SVD primarily affects the small arteries, which penetrate the white-matter substance and the leptomeningeal spaces, together with the perivascular spaces (PVS) and blood–brain barrier (BBB) [26,27,28,29,30]. The relevance of the SVD is crucial; it accounts for more than 25% of all lacunar events and is the cause of 45% of dementia cases in the cohort of people aged 80 and over [6,29]. SVD is often a silent form of subcortical-vascular burden, with a wide range of clinical manifestations, from small lacunar-ischemic events to large, confluent white-matter hyperintensities with a concomitant neuropsychological disruption called sVAD.

The primary gross pathology related to brain SVD is arteriolosclerosis, which is associated with the aging process, with the related loss of arterial elasticity, a decrease in the VSMCs’ activity, and inadequate support by the autonomic nervous system, the consequent impaired endothelium-mediated baroreflex activity, and cholinergic innervation [29,31]. Arteriolosclerosis occurs in two forms, hyperplastic and hyaline [32,33,34]. The former is the most-common lesion, evidenced by significant thickening, due to the concentric smooth-muscle-cell proliferation, with an “onion skin” deposition into the wall of the arterioles, obliterating the lumen [32,33]. Hyaline arteriolosclerosis is a progressive substitution of the smooth muscle cells by collagen, derived by the leakage of the plasma proteins and an enlargement of the basement membrane [35,36,37,38].

In addition, arteriosclerosis is tightly bound to a decrease in vascular compliance auto-regulation [39], which is even more significant in the deeper part of the brain structures (basal ganglia, pons, mesencephalon), which do not rely directly on the Willis flow but are powerfully supplied by small arteries [29]. Therefore, these alterations affect the highly regulated system of profound cerebral blood flow, and its disruption alters the “retrograde vasodilatation system” and therefore compromises neurovascular coupling [29].

Synoptically, arteriolosclerosis induces:

1. Chronic hypoperfusion in the brain’s deeper structure, inducing incomplete deep white-matter ischemia. Most frequently, the lesions affect the frontal and prefrontal-thalamus-basal forebrain networks [40]. Specifically, the lesions involve brain parts characterized by high metabolic rates, such as the insula, caudate nucleus, putamen, and the prefrontal and middle-frontal gyrus [29,41,42].
2. Consequent perivascular inflammation, with a progressive BBB disruption, occlusion of deep-draining veins, induces a severe leakage of albumin and plasma cells and significant alterations in the paraglymphatic system, localized in the PVS [43,44].
3. Astrocyte gliosis, diffuse demyelination, and disruption of axonal [45].
4. An impaired cholinergic vasoreactivity [45].

The induced and progressive BBB dysfunction is tightly related to an altered endothelium function, impaired vasoreactivity, and an altered paraglymphatic system [30]. The BBB leakage increases with aging and is associated with Alzheimer’s disease and lacunar strokes, and, more importantly, the amount of leakage correlates with the severity of white-matter hyperintensity [46,47]. Numerous studies in healthy animal models have shown that, usually, BBB permeability is extremely low with minimal passive leakage of plasma proteins, while animal models of various brain disorders, such as acute ischemia and chronic white-matter ischemia, are characterized by serious alterations in BBB function [46,47,48,49,50,51]. Moreover, BBB disruption is considered a result of white-matter inflammation, which is a consequence of chronic hypoperfusion in cerebral SVD [52], potentiating the continuation and prolongation of the white-matter sufferance [53,54,55]. In particular, the leakage of fluid and plasma components, such as proteins, into the perivascular tissues can cause vasogenic edema and inflammation, starting from the chronic hypoperfusion, and might promote the brain damage and thickening and stiffness of arteriole walls that lead to impaired vasodilation, and the compromised transport of oxygen and nutrients [46,47]. For example, after the leakage, a glycoprotein complex fibrinogen converts to fibrin and causes several deterioration processes, such as microglia activation and inflammation promotion [46,56]. Moreover, by binding amyloid β-peptide (Aβ), fibrin inhibits its clearance and helps the formation of amyloid-β plaque leading to pericyte loss, which is also common in Alzheimer’s disease [46,56]. Severe cerebral amyloid angiopathy (CAA) is commonly observed in SVD. In CAA, Aβ accumulates in arteries and arterioles walls, leading to VSMCs loss and fibrinoid necrosis, making them more prone to rupture microhemorrhages [29,49,50,51]. In addition, fibrin leads to demyelination and neuronal damage [46,56].

Furthermore, BBB disruption is somehow related, in brain SVD, to microglial activation that advocates the apoptosis. Markers of the chronic neuroinflammation process determined by BBB leakage are the increase in the concentration of caspase 3 RNA and matrix-metalloprotease 2 (MMP-2) expression [57]. The main consequence is directly found in a rapid mitochondrial dysfunction and an impaired endothelial response to vasodilators [58]. In addition, there is a down-regulation of the Rho-associated protein kinase (ROCK) [59]. ROCK and their associate proteins (ezrin, radixin and moesin) help maintain leukocyte adhesion molecule activity; their downregulation promotes endothelial inflammation without any control [29].

Chronic hypoperfusion and incomplete but progressive deep white-matter ischemia, consequent perivascular inflammation, amyloid deposition, the BBB disruption, the occlusion of the drainage veins, and the altered paraglymphatic systems, astrocyte gliosis, diffuse demyelination, axonal loss and an impaired cholinergic vasoreactivity, induce constant inflammation maintenance. The microglial activation (M1 preponderant), induced as the first response to the chronic hypoperfusion condition, occurs first in the hippocampus, then throughout the white matter and the thalamus, and at the end in the cortical areas [60,61,62,63]. At the beginning of the inflammation process, astrocytes react with primary consistent proliferation but with progressive degeneration and loss [62,63]. This event is one of the primary determinants of the dysregulation of the astrocytic control of the neuronal metabolic requests induced by ischemia and altered glutamate signals [64]. Their loss is a fundamental process inside the altered neurovascular coupling, which is a constant in brain SVD pathological stories [29,65].

Soon after, the other relevant alteration in brain SVD is a compromised response to the endothelium vasodilators, such as NO [66], prostacyclin [67], and endothelium-derived hyperpolarizing factors (EDHF) [68]. Mitochondrial dysfunction, associated with reactive-oxygen-species (ROS) overproduction, decreased endothelial NO synthase (eNOS) activity, and consequent low-NO bioavailability, contributes to the disruption of the cerebral endothelial cells and endothelial walls [69,70,71,72]. Decreased NO concentration in SVD represents a marker of compromised endothelial regulatory response against external factors such as hypercapnia, reducing the ability of the endothelium to properly proceed with neurovascular coupling to potentiate the correct metabolic requirement of cortical areas of the brain [70,73,74].

Finally, in SVD, an alteration in the cholinergic control that impairs the vasoregulatory properties of small vessels has been signaled by different studies and implemented by neuroinflammaging conditions [74,75,76]. This alteration can counterbalance the acetylcholine release, and the significant consequences are a decrease in vascular tone and BBB permeability associated with a loss of internal vascular remodeling [74,76].

Worth mentioning here is the recently recognized relationship between cerebral SVD and retinal microvascular dysfunction [77]. Under hyperglycemia conditions, the concentration of reactive oxygen species increases, eNOS activity is reduced and, consequently, the NO bioavailability which, as mentioned earlier, leads to endothelial dysfunction and diabetic atherosclerosis [77,78,79]. Moreover, persistent hypertension leads to remodeling of small arteries and impaired dilation of blood vessels [78]. Polyol pathway hyperactivity triggered by hyperglycemia is proposed as one of the possible mechanisms in the development and progression of both diabetic retinopathy and SVD, meaning that retinal-microvascular abnormalities are pathophysiologically associated with cerebral SVD [77]. Moreover, studies have shown that people with diabetes and microvascular retinal lesions are prone to brain microbleeds [80].

## 4. Useful Biomarkers in CMD and Brain SVD

Pathophysiological processes involved in CMD, such as inflammation, oxidative stress, and coagulation, have been thoroughly established, as well as active molecules. Therefore, in the context of endothelial-microcirculation dysfunction, these markers may be of paramount importance for its diagnosis and provide valuable prognostic insights.

**Figure 2:**
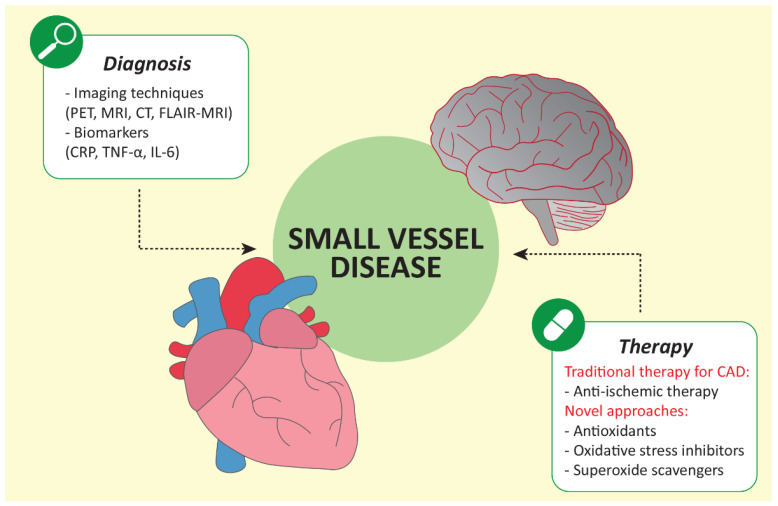
The figure describes diagnostic tools, both the imaging techniques and biomarkers for small-vessel disease, and the current therapeutic approach. CAD, coronary artery disease; CRP, C-reactive protein; CT, computed tomography; FLAIR-MRI, fluid-attenuated inversion recovery magnetic-resonance imaging; MRI, magnetic-resonance imaging; PET, positron-emission tomography; TNF-α, tumor-necrosis-factor α; IL-6, interleukin.

Pro-inflammatory mediators could be used as diagnostic and prognostic biomarkers for SVD because they alter endothelial function, modulating the immune response, oxidative processes, and coagulation. It has already been observed that C-reactive protein (CRP), interleukin (IL)-6, and tumor-necrosis-factor α (TNF-α) are elevated in patients with CMD and that higher CRP levels were associated with lower blood-flow reserves, both in the presence of obstructive CAD and without it (Figure 2) [2]. In addition, molecules such as uric acid, asymmetric dimethylarginine (ADMA), low-density lipoprotein, and homocysteine, that influence the bioavailability of NO, resulting in endothelial dysfunction, could be considered biomarkers for SVD. Several studies have already shown an increase in ADMA, uric acid and homocysteine in patients with CMD and a higher risk of adverse outcomes [2,81]. Regarding the balance of coagulation, it is worth noting that thrombomodulin and the von Willebrand factor are elevated in patients with CMD; therefore, they can be used as robust markers in the diagnosis [2].

Cerebral SVD can be characterized by decreased blood flow, ischemia and chronic white-matter inflammation, leading to various deterioration processes in the brain, such as axon damage, demyelination and astrocytes gliosis [45,57]. After ischemia, it has been shown that there is an increase in the expression of RNA caspase-3 and MMP-2 by activated microglia, molecules that could be suitable as diagnostic markers, providing insight into underlying processes [57].

Age-related mitochondrial dysfunction and cell senescence are mechanisms that, at a deeper level, explain the endothelium dysfunction in cerebral SVD [58,82]. The mitochondrial dysfunction leads to an increase in reactive oxygen spaces, reduced-eNOS activity and low-NO concentration [58]. Strong subsequent effects of mitochondrial oxidative damage on the endothelium and brain barrier cause a cascade of potentiating inflammatory events. It has been noted that SVD is present in all the basic mechanisms of inflammation that trigger neurodegeneration, such as apoptosis, necroptosis, neuronal autophagy, microglial activation, Wallerian degeneration, demyelination, and astrocytosis [83,84]. The peculiar phenomenon observed in SVD is necroptosis, a different form of programmed cell death which, unlike apoptosis, does not involve caspase activation yet involves the loss of plasma membrane integrity through the receptor-interacting serine/threonine-protein kinase 1 (RIPK-1) and the mixed lineage kinase domain-like (MLKL) [85].

As pointed out by studies on animal models, increased oxidative stress as a consequence of hypoxia, via reduced NO concentration, promotes further DNA and protein damage, impaired vascular function and tone, and results in compromised neurovascular coupling [86]. Nicotinamide adenine dinucleotide phosphate oxidases-2 (Nox2) plays a key role in the production of oxygen species, promoting BBB dysfunction; therefore, it can be used as a marker of adverse processes or as a therapeutic target [87].

As mentioned previously, endothelial dysfunction in the brain corresponds to an altered response to the endothelium-derived NO-vasodilators, prostacyclin, CRP, and EDHF. Therefore, those markers could be helpful as diagnostic and prognostic parameters. In addition, albumin extravasations are prevalent in brain aging and reflect BBB leakage and altered endothelial permeability. Another parameter, such as a higher cerebrospinal fluid/plasma albumin ratio, also indicates the plasma leakage and is associated with white-matter lesions in cerebral SVD [88]. In addition, additional markers of the latter that can be of great use are hypoxia-inducible factor 1-alpha, vascular endothelial growth factor receptor 2, and neuroglobin [89,90]. Lastly, markers such as IL-6, thrombomodulin and von Willebrand factor are worth mentioning here, which can be used to identify the presence of SVD both in the brain and heart [91,92,93]. More recent approaches have been debated more widely in a recently published work [52].

## 5. Diagnosis of CMD and Brain SVD

From a clinical perspective, CMD often represents a subtle and unrecognized entity, essentially being called into question when symptoms of myocardial ischemia such as chest pain or angina equivalents cannot be justified by any manifest cardiovascular or systemic pathology [94]. Similarly, abnormalities of brain microcirculation can be responsible for cognitive impairment and dementia, especially when neurodegenerative disorders such as Alzheimer’s and Parkinson’s diseases have been ruled out [95].

In the modern era, where the range of advanced diagnostic tools available to physicians keeps expanding, microvascular dysfunction can be extensively investigated and not only hypothesized, as in the past. Particularly, positron emission tomography (PET) and cardiac magnetic resonance (CMR) represent critical techniques for the non-invasive assessment of CMD through the evaluation of myocardial perfusion following vasodilator induction [96]. Different pharmacologic stressors such as adenosine and dipyridamole are employed to achieve maximal hyperemia [12], during which high-resolution perfusion CMR can demonstrate a microvascular dysfunction by quantifying the blood-flow distribution across myocardial layers [97]. Likewise, advanced magnetic resonance imaging techniques and PET scans can provide relevant clues to the presence and severity of cerebral SVD [98,99]. Traditionally, computed tomography (CT) imaging has been used to detect morphologic lesions without evaluating their functional consequences. However, this limitation has been overcome in the cardiovascular area, where CT angiography and CT-perfusion scanning combine with overlapping anatomical images with functional testing [100]. Specifically, static CT perfusion suggests a microvascular impairment in patients with anginous symptoms and no obstructive CAD when myocardial perfusion reserve in the subepicardial layer is significantly higher than in the subendocardial layer [101]. Furthermore, dynamic myocardial CT perfusion is an insightful diagnostic tool for CMD because of quantitative perfusion evaluation and myocardial blood-flow estimation [102].

Neuroimaging techniques are the primary tool for the neurologist to diagnose brain SVD correctly, and useful neuroimaging markers involve discrete lacunar infarcts, white-matter hyperintensities (WMHs), lacunes, enlarged PVS, and cerebral microbleeds [6,103]. WMHs, in comparison with the healthy brain, appear hyperintense and confluent on a T2-weighted (T2) MRI or fluid-attenuated inversion recovery (FLAIR) MRI [6,98,103]. The number of lacunes and confluent white-matter changes are associated with cognitive impairment’s severity [6,104]. The most definitive diagnosis is the final result of SVD, a confluent alteration in white matter, which causes the relevant sVAD. According to NINDS-AIREN criteria and the DSM-V R (Fifth Edition—revised), sVAD is diagnosed when the MRI scan shows moderate to severe ischemic white-matter alterations and one or more lacunar infarct [105,106,107,108]. In addition, numerous studies pointed out the correlation between cerebral impairment, brain SVD and arterial stiffness [109,110]. Arterial stiffness leads to microvessel arteriosclerosis and impaired endothelial function. Saji et al. proposed the ‘tsunami effect’ as an explanation for the augmented blood flow pulsatility as a consequence of the narrowing due to atherosclerosis and vascular stiffness towards the cerebral parenchyma resulting in the brain SVD [109,110]. A more recent study has determined a tight relationship between large-arterial stiffness and the severity of enlarged perivascular spaces and cerebral microbleeds inside the deep brain structures, such as the basal ganglia and centrum semiovale [111]. A recently published study focused on that relationship pointed out that augmented carotid intima-media thickness and carotid-femoral pulse-wave velocity are parameters that indicate the presence of cerebral white-matter hyperintensities. Moreover, it seems interesting that the adjusted cerebral-resistance index and brachial flow-mediated dilation time to peak were linked with the severity of cerebral white-matter alteration; the ongoing altered white matter is independent of age and gender [112]. At the same time, when producing post-occlusive reactive hyperemia, this parameter could be considered a target of microvascular reactivity. Time of post-occlusive hyperemia is related to the expansion, and the severity of white-matter alterations, together with ankle Brachial Pressure Index and urinary albumin excretion rate [112].

## 6. Therapeutic Approaches for CMD and for Brain SVD

In the complex and heterogeneous field of microvascular dysfunction, optimal treatment is still unknown. Despite microvascular dysfunction being an increasingly recognized disease, data regarding the optimal treatment of this condition are incredibly lacking. No randomized trials have investigated possible approaches to reduce adverse cardiac events in patients with CMD [113]. Therefore, traditional anti-ischemic and risk-reduction therapies validated for epicardial CAD are used in patients with microvascular dysfunction, even if this approach may not be effective in this setting [114]. No data support antiplatelet agents, including acetylsalicylic acid, for microvascular dysfunction. Ticagrelor has been proposed to have a possible role in protecting the microcirculation with an adenosine-related mechanism. However, clinical studies investigating the possible role of Ticagrelor in this setting are still ongoing [115,116]. Angiotensin-converting enzyme inhibitors and statins have also been suggested to help improve endothelial dysfunction and reduce oxidative stress in some registry studies; however, randomized trials are lacking [116]. Finally, in a small randomized trial, six months of L-arginine supplementation was shown to be effective in ameliorating symptoms, coronary blood flow, and endothelial function in patients with microvascular disease [117]. This beneficial effect of L-arginine supplementation may be due to its role in the production of NO, which contributes to the regulation of microvascular tone, and various indirect mechanisms such as the production of other vasoactive factors, namely, endothelin-1 [117]. Few new possible specific treatments for microvascular dysfunction have been proposed. One strategy could be to inhibit Rho-kinase to reduce the inflammation of coronary adventitia, which seems to correlate to coronary spasms [118]. Antonopoulous et al. have proposed to develop treatments stimulating the perivascular adipose tissue to produce factors such as adiponectin or hydrogen sulfide, which, thanks to their vasoactive and vasorelaxant factors, could be beneficial [119].

Regarding the brain SVD, a possible strategy for effective treatment might focus on the beneficial role of antioxidants on the small vessel endothelium and BBB. As mentioned previously, NO bioavailability is low in SVD. The antioxidant could repair the severely disturbed endothelial function [120,121]. However, there is a massive discrepancy regarding the antioxidant supplementation. Several small clinical trials proved that the administration of potent antioxidants such as vitamin C and E positively affected vascular function, whereas the results of large clinical trials have failed in this intention [120,121,122,123,124]. The reasons could be various, such as incorrectly conducted studies, inappropriate subjects, time of the treatment initiation, initial concentration, and, in the end, the fact that extremely high concentrations of vitamins C and E are required to compete with the superoxide and NO reaction [121]. In conclusion, further studies and clinical trials are needed to clarify this issue.

Another interesting approach would be the implementation of the ROS scavenger, tempol, already used in experimental studies and accepted to reduce oxidative stress [125,126]. In a similar context, a promising approach would be edaravone, an O_2_ scavenger with positive effects on the vessels, heart, and brain, due to its anti-inflammatory, anti-apoptotic and anti-necrotic properties [127].

Nox inhibitors are a promising therapeutic strategy for the brain SVD since Nox-mediated oxidative stress is well-established in this setting, particularly the primary contributor, Nox2. However, it could be discussed that prolonged selective therapies could have beneficial effects in terms of brain SVD prevention, but they also could lead to an immunosuppression condition and many other side effects derived from different other Nox [124,128,129,130]. In addition, the most effective and frequently prescribed drugs for cardiovascular treatment, such as angiotensin-converting-enzyme inhibitors, angiotensin II receptor type 1 antagonists, and statins, proved to inhibit Nox and therefore reduce oxidative stress [124,131,132].

Despite this sparse data, much has to be done to research novel therapies for the microvascular dysfunction in the heart and brain [113,133].

Finally, managing traditional risk factors such as high blood pressure, BMI, diabetes mellitus, insulin resistance and obesity is essential, as one of the approaches to preventing SVD. Regular physical activity and a healthy diet are other ways to prevent this disease, and most other diseases. It has been observed that individuals with higher salt intake in their diet were more susceptible to lacunar stroke, white-matter hyperintensity and microbleeds, indicating that salt reduction might lower the incidence of SVD [134]. Still, additional studies should confirm this finding. In addition, modulation of undesirable dietary patterns such as highly processed foods, high-fat intake, and restriction of animal products may also be helpful in reducing the likelihood of SVD development and progression [135]. The reason for this lies in the close connection between this type of food and deterioration processes such as arteriosclerosis, arteriosclerosis, diabetes mellitus and the creation of circulating microparticles with a role in inflammation, oxidative stress, microbiota dysbiosis, coagulation and microthrombosis [135].

## 7. Conclusions

In summary, impaired function of the endothelium of small vessels in the heart lead to ischemia independently of the presence of obstructive CAD, and it correlates with poor outcomes. SVD in the brain causes neurological issues such as stroke and dementia, which are very common among the older population. It is not surprising that, due to the aging of the population, the diagnosis of SVD has increased over the years, and it is assumed that the actual prevalence is even higher. However, it should be borne in mind that the epidemiology of SVD is less well established and that only recently have clinical reports begun to pay special attention to this disease.

Imaging techniques such as CT and MRI in cardiac and brain settings are of the utmost importance for accurate diagnosis and risk stratification. Inflammatory markers such as CRP, IL-6, and TNF-α have been increased in SVD, and they provide helpful insight into the severity of the disease.

Numerous molecules that are recognized to be involved in the reduction in NO bioavailability and the promotion of oxidative stress are the target of excessive investigations as diagnostic biomarkers and potential therapeutic approaches. Currently, accepted therapeutic approaches against SVD rely on anti-ischemic and risk-reduction therapies for epicardial CAD. In this context, it is crucial to mention antioxidants such as vitamin E and vitamin C, tempol, edavaron, and Nox inhibitors, which are already known for reducing oxidative stress and have attracted attention as potential therapeutic solutions. However, further laboratory experiments, clinical studies, and trials are needed to confirm these findings. For example, the currently available data regarding vitamin E and C in these settings have yielded inconsistent and confusing results for various reasons, such as incorrectly conducted studies and an inadequate cohort of subjects.

## Data Availability

Not applicable.
